# Multi-omics approaches for revealing the complexity of cardiovascular disease

**DOI:** 10.1093/bib/bbab061

**Published:** 2021-03-17

**Authors:** Stephen Doran, Muhammad Arif, Simon Lam, Abdulahad Bayraktar, Hasan Turkez, Mathias Uhlen, Jan Boren, Adil Mardinoglu

**Affiliations:** Centre for Host-Microbiome Interactions, Faculty of Dentistry, Oral & Craniofacial Sciences, King's College London, London, SE1 9RT, United Kingdom; Science for Life Laboratory, KTH - Royal Institute of Technology, Stockholm, Sweden; Centre for Host-Microbiome Interactions, Faculty of Dentistry, Oral & Craniofacial Sciences, King's College London, London, SE1 9RT, United Kingdom; Centre for Host-Microbiome Interactions, Faculty of Dentistry, Oral & Craniofacial Sciences, King's College London, London, SE1 9RT, United Kingdom; Department of Medical Biology, Faculty of Medicine, Atatürk University, Erzurum, Turkey; Science for Life Laboratory, KTH - Royal Institute of Technology, Stockholm, Sweden; Institute of Medicine, Department of Molecular and Clinical Medicine, University of Gothenburg and Sahlgrenska University Hospital Gothenburg, Sweden; Centre for Host-Microbiome Interactions, Faculty of Dentistry, Oral & Craniofacial Sciences, King's College London, London, SE1 9RT, United Kingdom; Science for Life Laboratory, KTH - Royal Institute of Technology, Stockholm, Sweden

**Keywords:** systems biology, cardiovascular disease, omics integration, integrated networks, genome-scale metabolic model

## Abstract

The development and progression of cardiovascular disease (CVD) can mainly be attributed to the narrowing of blood vessels caused by atherosclerosis and thrombosis, which induces organ damage that will result in end-organ dysfunction characterized by events such as myocardial infarction or stroke. It is also essential to consider other contributory factors to CVD, including cardiac remodelling caused by cardiomyopathies and co-morbidities with other diseases such as chronic kidney disease. Besides, there is a growing amount of evidence linking the gut microbiota to CVD through several metabolic pathways. Hence, it is of utmost importance to decipher the underlying molecular mechanisms associated with these disease states to elucidate the development and progression of CVD. A wide array of systems biology approaches incorporating multi-omics data have emerged as an invaluable tool in establishing alterations in specific cell types and identifying modifications in signalling events that promote disease development. Here, we review recent studies that apply multi-omics approaches to further understand the underlying causes of CVD and provide possible treatment strategies by identifying novel drug targets and biomarkers. We also discuss very recent advances in gut microbiota research with an emphasis on how diet and microbial composition can impact the development of CVD. Finally, we present various biological network analyses and other independent studies that have been employed for providing mechanistic explanation and developing treatment strategies for end-stage CVD, namely myocardial infarction and stroke.

## Introduction

Cardiovascular disease (CVD), specifically coronary heart disease and stroke, remain the leading cause of death worldwide accounting for a combined annual total of over 17 million deaths [1]. A significant number of these deaths are linked to chronic kidney disease (CKD) with ~8.5% of global heart disease deaths and ~8% of stroke-related deaths attributable to decreased kidney function [2]. Most CVD-related complications are caused by ischemic CVD [3, 4], which is associated with the accumulation of fat inside the arteries (atherosclerosis) coupled with an increased risk of blood clots (thrombosis). Consequently, the rupture of atherosclerotic plaques and the formation of blood clots are the dominant cause of myocardial infarction, heart failure and stroke [5, 6]. Besides, heart failure can also occur through non-ischemic CVD, characterized by adverse cardiac remodelling and cardiomyopathies that mainly occur through left ventricular cavity remodelling [7], thus further emphasizing the complexity of CVD. The progression of these CVDs can primarily be attributed to disturbances in both global and cardiac-specific metabolism [8]. Furthermore, specific CVD components are influenced by genetic and epigenetic factors, whether it be mutations causing adverse cardiac remodelling in heart failure [9] or modifications that affect the susceptibility of the arterial wall to plaque formation [10].

Although the prevalence of CVD continues to significantly increase worldwide, a detailed understanding of the molecular mechanisms associated with the underlying causes of CVD is still lacking in some areas, particularly for non-ischemic CVD. To rectify this, extensive efforts and resources have been placed into systems biology approaches, which attempt to further understand the underlying conditions of CVD to improve early detection and treatment of CVD. The recent advances in high-throughput technology have enhanced researchers’ ability to analyse whole genomes, transcriptomes, proteomes, metabolomes and metagenomes, thus facilitating the integrative analysis of multi-omics data to identify causal genes and reveal underlying molecular mechanisms that are involved in the progression of cardiovascular events [11]. In particular, the development of genome-scale metabolic models (GEMs) allows for describing molecular variation and quantifying flux through metabolic pathways in different physiological systems [12, 13]. To date, several GEMs have been developed to further understand the heart-specific metabolic processes in health and disease [14–16]. Moreover, this wealth of data has highlighted the diverse pathological aspects of CVD, which will be vital in facilitating risk stratification of patients and optimizing intervention to prevent CVD progression.

There is a growing body of literature supporting the notion that specific metabolites secreted by the gut microbiome can increase cardiovascular risk based on recent studies associating elevated levels of lipopolysaccharides (LPS) [17], gammabutyrobetaine [18], trimethyllysine [19] and uremic toxins including indoxyl sulphate, indole-3-acetic acid and P-Cresyl sulphate [20], with increased risk of CVD events. Most notably, there is a substantial amount of evidence linking elevated levels of the gut microbe-derived trimethylamine-N-oxide (TMAO) to CVD [18, 21–23] and CKD [24] through a mechanism involving oxidation of trimethylamine (TMA) to TMAO in the liver. The subsequent circulation of TMAO in the bloodstream can induce mechanistic changes in several different cell types including (i) increased levels of scavenger receptors in macrophages which alter the cell phenotype and result in the production of foam cells, (ii) increases in NFκB activation, adhesion proteins and NLRP3 inflammasome activation coupled with a decrease in nitric oxide production in endothelial cells (ECs) and (iii) platelet hyper-reactivity through increased levels of Ca^2+^ release. Furthermore, these studies showed that the microbial production of TMA is strongly dependent on dietary precursors, and hence it is a combination of microbial metabolism and dietary interactions that are involved in the development of CVD. It has also recently been shown that the gut microbiota composition can alter various serum metabolite concentrations that are significantly associated with coronary artery disease (CAD) severity [25]. Hence, integrating metagenomics with other omics approaches (i.e. genomics, transcriptomics, proteomics, metabolomics) to study host-microbiome interactions will allow for advanced panomics enabling deep phenotyping that could prove invaluable in developing precision medicine for the treatment of CVD [26].

In this review, we first summarize how systems biology approaches have been applied towards mechanistically explaining the different underlying causes of CVD including atherosclerosis, thrombosis, adverse cardiac remodelling and CKD. We mainly focus on studies that identify biomarkers for stratification and promote the development of personalized treatment strategies based on a patient’s unique phenotype. Secondly, we assess the current state of gut microbiome research in explaining CVD pathophysiology accounting for the effect of diet. Moreover, we review the application of multi-omics analyses to the gut microbiota and discuss identification of gut microbe-derived metabolites that have been proposed as biomarkers for major adverse cardiac events. Finally, we comprehensively review the application of integrated multi-omics systems biology methods for end-stage CVD mainly characterized by CAD and ischaemic stroke.

## Multi-omics approaches towards explaining the underlying causes of CVD

### Atherosclerosis

**Figure 1:**
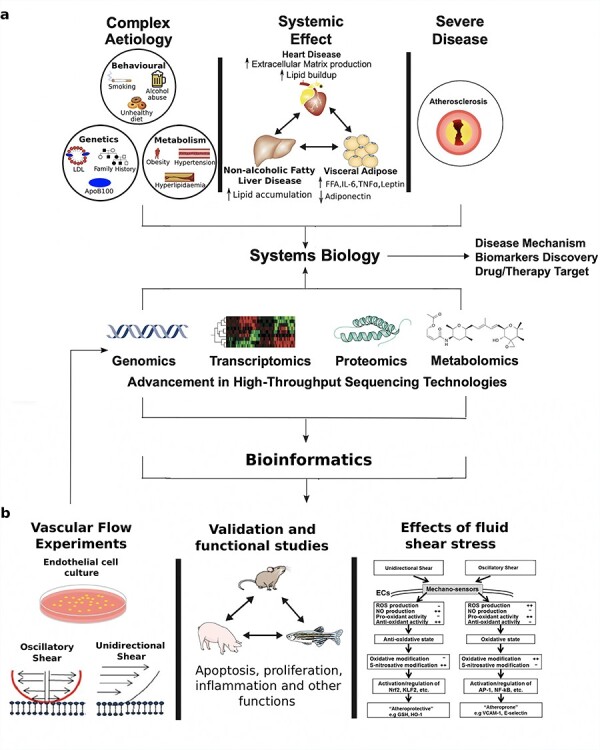
Schematic pathway for systems biology studies that utilize different omics data (genomics, transcriptomics, proteomics and metabolomics) to (A) further elucidate the complex aetiology and systemic effects arising from different tissues (heart, liver and visceral adipose) that contribute towards the development of atherosclerosis, and (B) investigate endothelial cell responses to flow and determine how different fluid shear stress states can promote either an ‘atheroprotective’ or ‘atheroprone’ state.

Atherosclerosis is characterized by artery blockages that arise from plaque deposits consisting of cholesterol, lipid proteins, cellular waste products and fibrin [27]. The prerequisite for plaque formation is the presence of low-density lipoprotein (LDL)-cholesterol, particularly in oxidized form [28]. Over the past 50 years, the progression of atherosclerosis has been linked to a multitude of risk factors, including hyperlipidaemia, behavioural, metabolism-related, and genetic risk factors, together with systemic changes in the body (Figure 1A). Previous research centred around the observation of related clinical variables and autopsy studies showed that behavioural risk factors, including physical inactivity [29, 30], unhealthy diet [31, 32], stress, and alcohol and tobacco abuse [33, 34], play a significant role in the progression of atherosclerosis. Moreover, the aforementioned risk factors cause alterations in metabolism [35–37] related to the onset of hypertension, obesity, diabetes and hyperlipidaemia, which are also risk factors for atherosclerosis. In addition, it has been shown that genetic factors [38, 39], such as elevated levels of apolipoprotein B (apoB)-containing lipoproteins and to a lesser extent increased levels of LDL and very-LDLs, are associated with the disease, together with increased blood pressure, decreased high-density lipoproteins levels and family history. Considering several risk factors exist for atherosclerosis, there has been an increased focus on discovering pleiotropic effects of variants in lipoprotein metabolism genes with a recent phenome-wide association study (PheWAS) identifying two LDL receptor variants as having several significant lipid-related phenotype associations [40]. Furthermore, several studies have connected atherosclerosis with systemic inflammatory changes in the body, such as the increase of pro-inflammatory proteins in adipose [41] and liver [42]. Specifically, in adipose tissue, it has been shown that an increase in free fatty acid levels led to the increase of multiple lipoproteins that cause lipid deposits in the arteries.

The complex aetiology and systemic effects of atherosclerosis, combined with its severity, has contributed towards its current status as one of the most studied diseases. Recent advancements in high-throughput sequencing and systems biology have allowed for a greater understanding of the underlying molecular mechanisms that contribute to atherosclerosis development and progression. As previously discussed, atherosclerosis is associated with genetic factors, and this has resulted in several genome-wide association studies (GWAS) with subsequent identification of multiple loci that are associated with atherosclerosis [43–45]. Moreover, studies with transcriptomics technologies have provided an overview of the global transcriptome map of the disease and revealed important candidate biomarker genes, biological processes, and pathways related to the disease in several species [46–49]. For example, an *in vitro* study detailing the profile and functions of long non-coding RNAs (lncRNA) and their association with atherosclerosis revealed the molecular mechanism surrounding ANRIL’s pro-atherogenic effect, which was validated in primary human cells from patients [50]. Furthermore, circular non-coding RNA of this same gene has been shown to induce processes that lead to apoptosis and inhibition of cell proliferation in human vascular cell and tissues, which are critical cellular functions closely related to atherosclerosis [51]. A separate study showed 180 differentially expressed lncRNA when comparing healthy samples with those from atherosclerosis-induced ischemic stroke patients, with SCARNA8 and SNRPN-2 being the most significant differentially expressed lncRNAs between the two sample types. In addition to tissue-level transcriptomics analysis, single-cell maps of atherosclerotic plaques have recently been built [52].

Other omics approaches, including proteomics [53, 54], lipidomics [55, 56] and metabolomics [57, 58], have been leveraged to provide overall disease profile and reveal possible early-stage biomarkers that can be used to further our understanding regarding the molecular mechanism of the disease (Figure 1A). This includes a very recent proteomics-based assessment which identified NPC2 and IGFBP7 as novel candidate biomarkers for atherosclerotic aortic aneurysm in humans [54]. In addition, a large-scale metabolomics study revealed 42 significantly altered metabolites in coronary atherosclerosis patients compared to controls, with nine of these metabolites combinatorically identifying the early stage of the disease with high accuracy (AUC = 0.890) [58] and belonging to a phospholipid, fatty acid, fructose and fucose, and tryptophan metabolism.

The continuous frictional force of blood flow across the vascular endothelium surface results in hemodynamic stress and pressure changes throughout each cardiac cycle. This interaction between the endothelium and fluid shear stress (FSS) is essential in maintaining vascular homeostasis and in protecting against or promoting atherosclerosis. Sustained unidirectional laminar shear (US) forces upregulate expression of genes and proteins in the endothelium that are protective against atherosclerosis. In contrast, oscillatory shear (OS) forces result in atherogenesis through the upregulation of EC genes and proteins that promote oxidative conditions and inflammatory states in the artery wall [59–61]. It is also now commonly accepted that FSS induces intimal hyperplasia and increased retention of subendothelial accumulation of apoB-containing lipoproteins [62, 63].

The study of EC mechanobiology mainly takes place through *in vitro* experiments using techniques such as microarray [64] and next-generation sequencing [65], thus enabling a global view of the multiple signalling events occurring in response to US or OS (Figure 1B). For example, experimental data gleaned from a systematic time-series RNA-sequencing dataset was used for pathway analysis and construction of tissue factor (TF)-to-gene networks with the results providing insights into the dynamics of functional response of ECs to shear stress over time [65]. This included upregulation of many genes in several pathways involving inflammation, oxidative stress, cell cycle and angiogenesis, with initiation and termination of pathway upregulation occurring at different time points. For example, several early response genes involved in inflammation (*IL8, MCP-1, SELE* and *VCAM1*) showed changes during 2–9 h following the activation of TF nuclear factor-κB (NF-κB) by upregulation of genes contributing to reactive oxygen species production by 2 h. Hence, this combined gene-TF-phenotypic network was able to identify transcriptional regulation mechanisms for cellular functions that lead to defined endothelial phenotypes. Consequently, this provided insights into the causality of mechanisms leading to stress responses.

Further high-throughput, omics-based approaches include studies of shear stress regulation of the endothelial proteome [66] and DNA methylome [67]. Interestingly, flow-regulated proteins included several transforming growth factor-β (TGFβ) superfamily members, angiopoietin 2 (ANG2), vascular endothelial growth factor (VEGF) [68] and Notch receptors [69]. A previous PheWAS also found that a single nucleotide polymorphism in the *NOTCH2* gene was associated with increased risk of hypertension [70]. The regulation of genome-wide DNA methylation patterns by blood flow has been shown in pig ECs with differentially methylated regions being associated with oxidative stress, thus implicating this process in early-stage atherogenesis [67]. Flow has also been shown to alter the methylation of homeobox family genes in both mouse and pig models [67, 71]. To summarize, these different omics studies have revealed that FSS regulates various molecules that control diverse physiological processes, hence providing invaluable clues to the molecular mechanisms underlying atherosclerosis.

The large data sets produced by omics approaches can also be integrated to increase the power towards discovering novel molecules and pathways, such as the integration of microarray flow data with a bisulfite sequencing genome-wide methylation array, which identified genes downregulated by OS and containing hypermethylated promoter regions [71]. Of the identified genes, *HOXA5* and *KLF3* encode TFs containing cAMP response elements, supporting the notion that these loci’s methylation status could be acting as a mechanosensitive master switch in gene expression. These novel findings demonstrated that OS controls epigenomic DNA methylation patterns, which alters endothelial gene expression and promotes atherosclerosis. Future studies should explore the dynamics of proteins from distinctly expressed genes in shear, which would likely produce novel insights into mechanisms of atherosclerosis. Current limitations in the field:

### Platelet signalling and thrombosis

In addition to ECs and circulating coagulation proteins, platelets are crucial mediators of thrombosis and vascular haemostasis with the latter process maintaining regulation of blood flow and vascular integrity. Pathological factors may overwhelm normal haemostasis, leading to uncontrolled clot formation and blood vessel occlusion [72]. Traditional epidemiological studies identified several arterial thrombotic risk factors, including hypertension, high LDL-cholesterol levels and smoking. Family cohort studies have also identified hereditary thrombophilia as an additional risk factor associated with an increased risk of arterial thromboembolism [73].

Furthermore, a meta-analysis of cohort studies revealed that mean platelet volume (MPV) might be a useful prognostic marker in patients with CVD with elevated MPV values found in patients with acute MI or coronary angioplasty [74]. The human platelet proteome contains ~4000 unique proteins with the majority of these defined in a protein interaction network (PlateletWeb) [75] consisting of 13 652 interactions and 1704 detected phosphorylation events. Besides, a platelet GEM [76] has been constructed to aid further understanding of pathophysiological conditions through data-driven, systems analysis of platelet metabolism. Numerous genetically modified mouse models have been previously developed to explore genotype/phenotype linkages with regards to clot formation with defects in various platelet receptors, and membrane proteins (e.g. GPVI [glycoprotein VI] and P2Y_12_ [P2Y purinergic receptor, type Y12]) found to slow and reduce clot growth whilst enhancing embolization [77]. There have been several applications of transcriptomics approaches towards understanding platelet responsiveness, including a previous study which examined individual heterogeneity in platelet response to ADP and collagen-related peptide with 63 different genes being implicated in influencing platelet responsiveness [78]. Furthermore, an association study of several of these genes showed a putative association with myocardial infarction for *COMMD7* (COMM domain-containing protein 7) and a highly significant association for *LRRFIP1* (leucine-rich repeat (in FLII) interacting protein 1). Both genes were positive regulators of thrombus formation with proteomics analysis indicating *LRRFIP1* functions as a component of the platelet cytoskeleton and interacts with actin-remodelling proteins Flighless-1 and Drebrin [78]. A separate population study [79] assessing platelet hyperresponsiveness to arachidonic acid, ADP and PAR1/4-activating peptides through integrated plateletomics revealed many mRNAs and microRNAs (miRNA) which were differentially expressed by age and gender. Networks of miRNAs targeting mRNAs were identified for both categories, and the inverse relationship in these RNA pairs suggested that miRNAs regulate mRNA levels based on age and sex, hence implying that future platelet RNA association studies must account for these variables.

The multiscale and hierarchical nature of thrombosis requires a systems biology approach that can describe platelet and plasma function through events including platelet membrane receptor function, intracellular signalling and assembly of extracellular processes occurring in plasma or on platelet membranes [80]. There have been successful attempts *in silico* to model platelet signalling through kinetic models, including a full bottom-up model of platelet intracellular metabolism that incorporated phosphoinositide and calcium regulation [81]. This model accurately predicted steady-state resting concentrations and transient increases in intracellular calcium and inositol trisphosphate in response to ADP. Furthermore, several kinetic models of protease cascades have been developed to predict blood coagulation, the most widely used being the Kuharsky–Fogelson model [82] which considers platelet activation, deposition and mass transfer of reactive species. This model was also able to confirm the exceptional sensitivity of coagulation to initial conditions of tissue factor levels through experimentally validated [83] predictions of threshold concentrations of surface tissue factor that triggered clotting in human blood.

**Figure 2:**
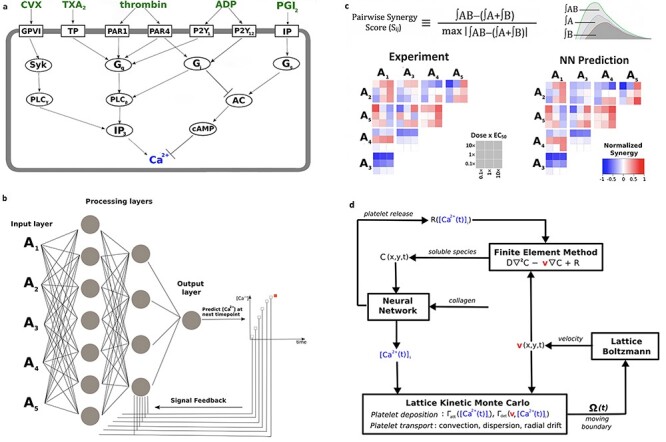
(A) Several signalling pathways in platelets converge on intracellular calcium release. (B) A dynamic neural network is used to train platelet response to combinatorial agonist activation. An input layer consisting of agonist concentrations is introduced to the 2-layer network at each time point and processing layers integrate input values with feedback signals for prediction of [Ca2+]i at the next timepoint. (C) Pairwise agonist synergy scores reflect gains or losses in calcium response due to agonist cross talk and are calculated for both experimental and predicted time-course traces to assess efficiency of the neural network. (D) Multiscale model of platelet deposition and thrombus formation under flow—this requires simultaneous solution of (1) intracellular platelet state ([Ca2+]i) and release reactions (R) for ADP and TXA2 calculated by Neural Network, (2) the instantaneous velocity field over a complex evolving platelet boundary □(t) calculated by Lattice Boltzmann, (3) concentration fields of ADP and TXA2 calculated by Finite Element Method, and (4) all platelet positions and attachment/detachment by Lattice Kinetic Monte Carlo.

The aforementioned bottom-up signalling approaches were applied to individual receptor pathways and did not address signalling through combinatorial and time-dependent activators. Hence, another approach is required to capture the full process of platelets forming a core region through interaction with thrombus and collagen, with subsequent platelets in the growing thrombus shell being regulated by ADP and thromboxane signalling [84]. This integration of these numerous signals in human platelets (Figure 2A) was captured using a high-throughput assay that measured intracellular calcium levels in response to pairwise agonist scanning (PAS) and predicted cellular signalling responses to combinatorial stimuli through the training of a neural network (NN) model (Figure 2B) for prediction of entire platelet response space [85] (Figure 2C). This NN model could then successfully be applied to predict responses to sequential additions of agonists and ternary stimulation with experimental validation in a clinical setting confirming predictions such as a high-dimensional thrombotic risk at high thromboxane A2/prostaglandin I2 ratio consistent with the known cardiovascular risks of COX2 inhibitors [86]. The PAS approach in this study was limited to six agonists and further expansion of this PAS set would be required to map a major portion of the entire platelet response space by including agonists such as epinephrine and nitric oxide donors.

A more complete systems biology analysis of blood clotting requires comprehensive models which must account for extreme sensitivity to initial conditions, strong autocatalytic feedback and non-linearity of kinetic rates in order to successfully predict system outcomes that lead to thrombosis [87]. Patient-specific NN models of platelet activation allowed for larger scale simulations of thrombosis under flow through embedment of the NN model into a multiscale model [88] (Figure 2D) which incorporated three further sub-models: lattice Boltzmann, finite element method and lattice kinetic Monte Carlo [89]. These sub-models mentioned above accounted for changes in blood flow with increasing platelet deposition inside the blood vessel, the release of soluble platelet agonists (ADP and TXA_2_) and the platelets’ motion within the blood flow, respectively. This enabled multiscale prediction of donor-specific clotting events under different flow and pharmacologic modulation with a predictive accuracy of the simulations validated through real-time platelet deposition measurement using microfluidic devices. These findings offered a crucial breakthrough in describing how a myocardial infarction would progress based on a patient’s unique platelet phenotype. It was the first multiscale model approach that made donor-specific predictions of platelet function under flow in the presence of agonists and pharmacologic modulators of clinical relevance. For example, one of the study donors was insensitive to the COX-1 inhibitors aspirin and indomethacin under flow conditions with this trait exactly predicted in the multiscale modelling. Future studies should continue on this focus of developing tools to define platelet variations between patients and stratify them according to risk as determined by the relationship of platelet phenotype to prothrombotic events.

### Cardiac remodelling

Mechanical changes in myocardial cells and the myocardial dysfunction that arises from such changes can activate left ventricular cavity remodelling serving as a prelude towards heart failure and ultimately multi-organ damage. The application of systems biology in this area is essential in defining the early mechanical stresses and relevant functional modules to elucidate ventricular remodelling and the complex pathophysiology of heart failure [90]. The most common type of ventricular remodelling is dilation of the left ventricle with the associated disease termed dilated cardiomyopathy (DCM) and origins of cause not yet known. Hypertrophic cardiomyopathy (HCM) is a genetic disease that causes the left ventricle walls to become thicker and contract harder through mutations in contractile sarcomeric proteins. The established causal genes for HCM, including *MYH7* (myosin heavy chain 7) and *MYBPC3* (Myosin Binding Protein C3), account for ≅60% of HCM cases meaning the causal genes have not yet been identified in ≅40% of HCM patients [91]. A multitude of systems biology approaches have been employed to further understand the underlying molecular mechanisms of these two conditions.

**Figure 3:**
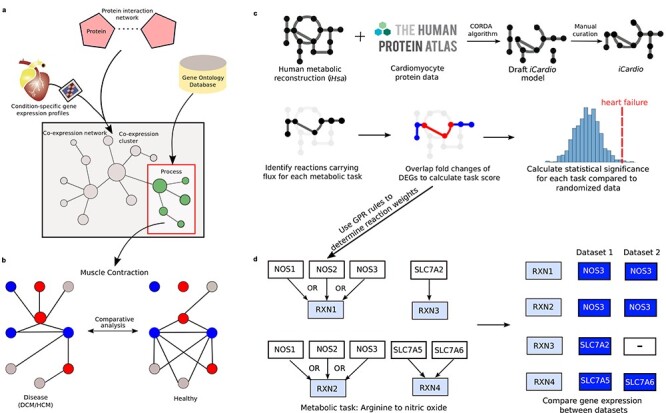
The integration of omics data and application of systems biology approaches towards providing mechanistic insights on heart failure. (A) Formation of a condition-specific co-expression network through integration of gene expression profiles with protein–protein interaction data and biological function annotations enable the identification of functional modules representative of different biological processes which are relevant towards the progression of heart failure. (B) A comparative analysis between identified functional modules can reveal dynamic variations in the modules between healthy and disease states which can be used to classify normal and disease samples, thus implying plausible molecular mechanisms that are involved in the progression of heart failure. (C) Construction of a cardiomyocyte metabolic model (iCardio) through integration with protein data and manual curation from metabolic tasks. Functional metabolic changes are identified from gene expression data using metabolic tasks. (D) The application of GPR rules in determining reaction weights based on gene expression data, thereby assigning the expression of one gene as governing the reaction. The selected genes can differ between different datasets while producing the same net result, as seen here with a statistically significant decrease in the conversion of arginine to nitric oxide.

There have been several studies towards explaining the aetiology of DCM, including a recent transcriptome analysis of human heart failure that revealed dysregulated cell adhesion in DCM [92]. Additionally, the use of animal models has provided new insights into mutations responsible for the onset of DCM, including a mutation in the Integrin-linked kinase gene in zebrafish [93] that was later described in patients with DCM [94]. Another successful application of animal models in this area includes a time-course mouse model of DCM which showed perturbations in apoptosis, integrin and MAPK (mitogen-activated protein kinase) signalling at the early- and mid-disease stage of DCM through global proteomic profiling and enrichment maps [95]. Further human-based studies have employed network-based comparative approaches [96, 97] that integrated DCM related gene expression profiles with protein–protein interaction data and biological function annotations to produce condition-specific co-expression networks (Figure 3A). A comparative analysis was then employed to extract DCM exclusive subnetworks that could be used to classify normal and disease samples (Figure 3B). This approach revealed dynamic functional modules corresponding to muscle contraction and organ morphogenesis that are closely related to heart failure and hence provided new insights into the cause of DCM.

The diverse clinical and pathologic phenotypes in HCM can be partially explained by the upregulation of various genes [98]. Population heterogeneity undermines efforts to uncover significant HCM-associated genes, and this has recently been addressed using a personalized multi-omics approach that identified *Hes1* (hairy and enhancer of split-1) as a regulator of cardiac hypertrophy [99]. In an analogous manner to DCM, the complex phenotype associated with HCM is only partly explained by individual genetic variants, and more recent studies have focused on proteomic analysis of human myocardial tissue to explore the postgenomic phenotype [100]. The myocardial proteome of HCM revealed dysregulation of structural and metabolic proteins and raised lumican levels in HCM hearts, which provides further insight into the myocardial fibrosis characteristic of this disease. This finding was in agreement with an earlier study that utilized mouse models to show increased lumican production altering molecules essential for cardiac remodelling and fibrosis in cardiac fibroblasts [101].

Metabolic alterations that induce changes in substrate utilization have also been noted in diseased states associated with cardiac remodelling, such as the remodelling of glucose metabolism preceding pressure overload-induced HCM [102]. A recently developed kinetic model, *CardioGlyo*, was utilized to study the metabolic control of myocardial glycolysis and mathematical simulations revealed that reduction of phosphoglucose isomerase (PGI) activity directly affects myocyte growth through glucose-6-phosphate (G6P) accumulation, which is correlated with increased activation of the mTOR (mammalian target of rapamycin) signalling pathway that is involved in structural remodelling of the heart [103]. This association may be explained by G6P being redirected into the pentose phosphate pathway upon PGI inhibition, thus generating high amounts of NADPH (nicotinamide adenine dinucleotide phosphate, reduced), which has been linked to mTOR activation [104, 105]. A more comprehensive overview of metabolism in cardiomyocytes has been captured in models [14, 15] that utilized the global human metabolic network, termed Recon1, as a template when building cardiomyocyte-specific models. However, human models have greatly expanded since Recon1 to enable a more comprehensive description of human metabolism, and this has been captured in a recently published human GEM, termed *iHsa* [106], which was built in parallel as an expansion of the Human Metabolic Reaction 2.0 database [107]. This GEM offered the opportunity to create a more comprehensive cardiomyocyte-specific metabolic model (Figure 3C), termed *iCardio* [108], built using *iHsa* and data from the Human Protein Atlas (HPA) [16]. The model was integrated with multiple heart failure omics datasets to identify standard shifts in metabolic functions that are associated with heart failure, and this approach identified decreased NO (Figure 3D) and Neu5Ac synthesis as common metabolic markers of heart failure across transcriptomics datasets [108]. The GEM approach can also be utilized to identify causal factors for cardiac remodelling whether it is loss of function mutations in cardiovascular-related genes altering metabolic fluxes [109] or different diets leading to profound changes in the cardiac mitochondria, which may ultimately result in cell damage and heart failure [110].

### Chronic kidney disease

The high comorbidity of CKD and CVD has been well established with a substantial number of CKD mortalities having registered cardiovascular problems; whilst CVD mortalities in stage 3 and stage 4 CKD patients are 2-fold and 3-fold higher than controls, i.e. patients with normal renal function, respectively [111, 112]. Both diseases also share numerous common risk factors, such as smoking, hypertension, abnormal mineral metabolism and vascular calcification, increased inflammation, sympathetic nervous system overactivity, hypertension, obesity and diabetes mellitus [113–115]. In particular, hypertension may occur in the early stages of CKD and lead to the onset of more severe conditions.

**Figure 4:**
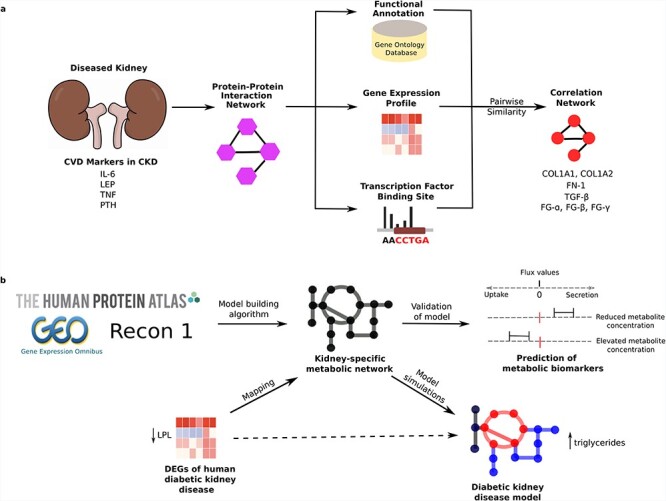
(A) Overview of a systems biology approach that links singular biomarker candidates towards deriving functional dependencies among chronic kidney disease (CKD) and cardiovascular disease (CVD). A set of genes associated with CVD in patients with CKD is used to construct a network of interacting proteins using reference data on known protein interactions. The functional interplay between interacting proteins was estimated by linking properties reflected by gene ontology terms, gene expression data characterizing CKD and TF binding sites with this methodology enabling detection of highly connected subnetworks associated with CKD and CVD. (B) The application of a kidney-specific metabolic model in determining metabolic process that are upregulated and downregulated in diabetic kidney disease resulting in the uptake and secretion of metabolites which may have an effect on the development and progression of CVD. Construction of the model was achieved through integration of proteome data from the Human Protein Atlas, transcriptome data from the Gene Expression Omnibus and application of a model-building algorithm that utilized Recon1 as a template to select only genes that were relevant to kidney.

Several systems biology studies have shown a system-wide linkage between the kidney, heart and other internal organs. For example, an earlier study applied an interactome analysis approach (Figure 4A) and identified leptin, tumour necrosis factor, interleukin-6 (IL-6) and parathyroid hormone as candidate biomarkers for CVDs and bone metabolism disorders in CKD patients [116]. Besides, collagens (COL1A1, COL1A2), fibronectin, TGF-β or components of fibrinogen (FG-α, FG-β, FG-γ) were components of highly connected subnetworks of proteins associated with CKD and CVD. A separate systems biology approach investigated the role of fibroblastic growth factor (FGF23) in CKD and on the cardiovascular system, including possible crosstalk with the renin-angiotensin system (RAS) [117]. This study was motivated by previous findings that identified increasing circulating levels of fibroblastic growth factor 23 (FGF23), which regulates phosphate and bone metabolism, as a decisive independent risk factor for both CKD and CVD [118]; whilst the RAS is activated in CKD [119] and contributes to increased cardiovascular mortality and renal failure progression. Ingenuity Pathway Analysis performed on genes in the FGF23 and RAS endocrine networks showed that multiple interconnections exist between the respective networks [117].

The most common cause of CKD is diabetes meaning the linkages between kidney and heart involving energy metabolism should be considered [120]. For example, plasma mannose levels have been associated with type 2 diabetes (T2D), CVD, and the onset and progression of diabetic kidney disease [121, 122]. A comprehensive human kidney metabolic model was built [123] by integrating proteome data from the HPA [124] with transcriptome data from the Gene Expression Omnibus [125] and applying a model-building algorithm [126, 127] that used Recon1 as a template to select only those genes that are relevant to kidney (Figure 4B). This model showed upregulated metabolic processes in the kidney that are tightly linked to diabetes, including energy metabolism, extracellular transport and lipid metabolism. Furthermore, flux variability analysis of differentially expressed genes for diabetic kidney disease revealed several essential genes in the disease process. This included downregulation of lipoprotein lipase, an enzyme that breaks down triglycerides [128], with the strong possibility of this observation being partially responsible for elevated levels of circulating triglycerides conferring increased risk of CVD. Besides, there was upregulation of nicotinamide N-methyltransferase, an activity change associated with obesity and T2D [129]. More recently, an alternative molecular process model was developed to predict the progression of renal function decline in T2D patients using systems biology-derived biomarkers [130]. This approach identified a small set of serum protein biomarkers that enhanced the prediction of renal function loss in such patients.

Future work should continue to focus on gaining further understanding of the ‘kidney-heart’ axis through a systems biology approach that posits circular communication loops amid the heart and kidney with the notion that perturbation at any level can propagate dysregulation throughout the circuit. It may also be beneficial to include the gut microbiome in this axis owing to the importance of the gut as a potential contributor to CKD and CVD-related complications [131], the latter of which we discuss in much more detail in this review.

### Role of the gut microbiota in CVD

The gut microbiota has emerged as a novel regulator of cardiovascular function and disease. Dysbiosis in the gut microbiome increases circulating microbial metabolites and bacterial structural components that may modify the function of metabolically relevant tissues and facilitate the development of CVD [132]. In particular, gut dysbiosis has been implicated in the pathology of numerous disturbances to the cardiovascular system, including atherosclerosis [133, 134], thrombosis [135], arterial hypertension and vascular dysfunction [136]. There are a variety of external factors that affect the composition and function of the gut microbiome, and these include most of the major risk factors for CVD, i.e. ageing, obesity, an inactive lifestyle and certain dietary patterns. From these external factors, the dietary pattern is widely accepted as one of the most critical factors in determining gut microbiota composition and function [137–140]. Hence, several studies have investigated the effects of dietary patterns in altering the gut microbiome and modifying signalling events that mediate CVD downstream of gut dysbiosis.

**Figure 5:**
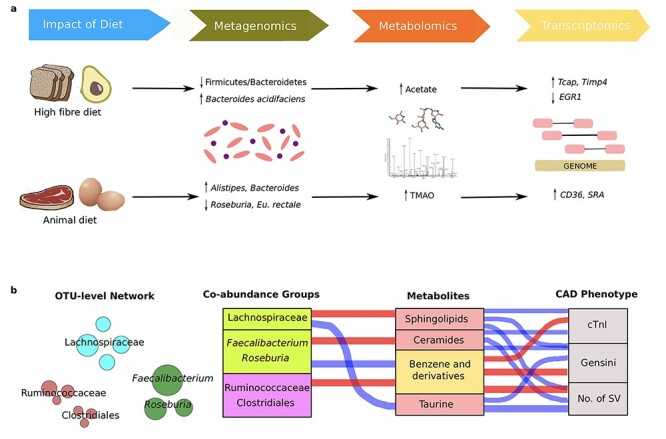
(A) The application of different fields of study (metagenomics, metabolomics and transcriptomics) in assessing how the impact of diet can alter the composition of the gut microbiota, plasma metabolite concentrations and expression of genes which protect against or promote biological processes related to cardiovascular disease. (B) Investigating the interrelationship between gut microbiota composition, host metabolic profile and main CAD phenotype. The OTU-level network indicates the abundance of each OTU in a CAG based on node size. Specific CAGs that are mainly composed of certain OTUs correlate with serum metabolite concentrations which in turn correlate with parameters representing CAD severity. Red connections indicate a positive correlation and blue connections represent negative correlations according to Spearman correlation test (FDR < 0.05). In the CAG column, the green boxes indicate CAGs that were highly enriched in the control group and the purple box represents a CAG that was increased in the severe CAD group. In the metabolomics column, the pink boxes represent CAD-negative metabotypes and the yellow box represents a CAD-positive metabotype.

There is evidence that dietary interventions may improve cardiovascular health and this has been confirmed through the application of multi-omics approaches (Figure 5A). For example, a diet high in fibre was associated with changes in the gut microbiota that protected against CVD development, including a decreased ratio of Firmicutes to Bacteroidetes, and an increased prevalence of *Bacteroides acidifaciens* [141]. The favourable effect of the high-fibre diet was also explained by an increased generation and distribution of acetate, with this short-chain fatty acid affecting several molecular changes that are associated with improved cardiovascular function. Besides, the cardiac transcriptome revealed that a high-fibre diet or acetate supplementation resulted in the upregulation of genes (*Tcap* [titin-cap] [142] and *Timp4* [tissue inhibitor of metalloprotease 4] [143]) that are strongly considered to have a preventive role for heart disease; whilst *EGR1* (early growth response protein 1), a master cardiovascular regulator [144–146], was downregulated in both the heart and kidney. Collectively, this data suggests that acetate production is an essential mechanism for regulation of cardiovascular function by the gut microbiota, and provide mechanistic insight into how dietary fibre protects against the development of CVD. These findings are supported by increasing evidence that insufficient consumption of dietary fibre results in a loss of bacterial species in the gut microbiome [147].

Alternatively, the effects of an animal-based protein diet have also been examined through multi-omics analyses (Figure 5A). Here, multiple studies [148–150] revealed that animal-based protein consumption affected the gut microbiota by increasing the counts of bile-tolerant anaerobes such as *Alistipes* and *Bacteroides*. There have been links between *Alistipes* and CVD risk factors such as hypertension, with a positive correlation observed between systolic blood pressure and the abundance of several species belonging to the *Alistipes* genus [151]. However, a number of studies have also indicated a protective role for *Alistipes* in CVDs with a drastic decrease of this genus in the intestinal tract of patients with atrial fibrillation [152], atherosclerotic CVDs [153] and congestive heart failure [154]. Hence, it is still unclear if associations with *Alistipes* are protective, beneficial or pathogenic. This exposes a severe limitation of taxon-based analysis, which overlooks variations of bacterial strains belonging to the same taxon. A separate study showed that a high protein/low carbohydrate diet resulted in reduced levels of *Roseburia* and *Eubacterium rectale* in the gut microbiota of subjects, accompanied by a decreased proportion of butyrate in their faeces [155]. In addition, several microbial genera that are promoted by the intake of red meat were associated with increased levels of TMAO [156], thus increasing the risk of CVD through upregulation of scavenger receptors including *CD36* (cluster of differentiation 36) [22, 157] and *SRA* (scavenger receptor A) [22]. These data support the established link between red meat consumption and CVD risk attributed to the high concentration of TMAO-producing nutrients, such as choline, phosphatidylcholine and L-carnitine, all of which are present in high concentrations in an animal diet including red meat, fish, milk and eggs.

Although several correlations have been established between CVD and the gut microbiota, it is much more challenging to identify potential causal relationships. An interdisciplinary approach is required to further understand gut bacterial-mediated mechanisms. Recent efforts have focussed on establishing causal relationships between the gut microbiota and CAD development via interactions with the host metabolic pathways. One such study revealed several alterations in gut microbial functional modules in CAD patients representative of amino acid transporters, LPS biosynthesis, the phosphotransferase system, vitamin metabolism, and the activities of short-chain fatty acids and TMA lyases [153]. These findings were in partial agreement with a separate study [154], which identified an elevation in microbial genes for LPS biosynthesis, TMAO and tryptophan generation in patients with chronic heart failure. This is of particular importance when considering the primary source of LPSs is the gut/gut microbiome, which provides an explanation for the greater plasma LPS levels in heart failure [158].

In addition to distinguishing between a CAD and healthy phenotype, the integration of multi-omics network analyses revealed that the composition of the gut microbiota and level of metabolites were significantly associated with CAD severity [25]. It was shown that several bacterial co-abundance groups (CAGs) might affect atherosclerosis by modulating several metabolic pathways in the host, which are positively or negatively associated with the CAD phenotype (Figure 5B). The basis for this approach is that key members of a CAG either thrive or decline together in response to a changing physiological environment and hence exist as functional groups [159] For example, a CAG that was enriched in the healthy control group, mainly composed of *Faecalibacterium* and *Roseburia*, was positively associated with ‘CAD-negative associated’ ceramides and negatively associated with ‘CAD-positive associated’ benzene derivatives. In contrast, a CAG mainly composed of operational taxonomic units from the Ruminoccocaceae family was higher in patients with CAD, which may partially be explained by the positive association between this CAG and the presence of benzene derivatives in the host metabolic profile. Hence, this CAG-based approach enabled the identification of functionally essential members of the gut microbiota in CVD and is recommended to be employed for further studies in place of the more conventional taxon-based analysis when investigating the links between the gut microbiota and the development of CVD. Future studies should focus on developing predictive systems-level approaches for each of these CAGs to elucidate causalities and quantify interactions between host, microbes and diet. In addition, this CAG approach could be used to investigate the influence of the microbiome on the plasma proteome with particular focus on signalling and inflammatory proteins pertinent to the cardiovascular system.

## Biological network analyses of end-stage CVD cohorts

### Coronary artery disease

CAD is the most common cause of CVD-related death and is caused by the accumulation of plaques in the coronary arteries, more widely known as atherosclerosis. Similar to atherosclerosis, CAD has a wide range of risk factors, including behavioural, metabolism-related and genetic risk factors. Patients diagnosed with CAD have also shown to be more prevalent to other chronic diseases, such as CKDs and end-stage renal diseases [160]. Statins are the most commonly used drugs used to treat CAD, especially in the early phase, due to their property of LDL reduction and their capability to decelerate the inflammation process [161] as demonstrated through various clinical trials (Table 1). They have also been shown to be beneficial in treating post-surgical CAD patients, both for cardiac stent and artery bypass graft patients [162–164].

**Table 1:** Survey of completed studies investigating different causes and treatments for coronary artery disease

Claim	Method	Result	Ref
Statins reduce LDL and are beneficial for post-surgical patients	Clinical trial	Rosuvastatin reduces LDL level by 50% and hCRP by 37%	[189]
	Clinical trial	Group with pre- and post-operative statins has significantly lower in-hospital deaths compared to placebo group (1.9% versus 6.91%, *P* = 0.002)	[162]
	Clinical trial	Risk of death rate was lower for statin treated patients by 54%	[164]
Genomic analysis reveals heritability and genetic variance of CAD	GWAS	Susceptibility to death from the disease was higher in male twins compared to female twins (0.57, 0.38, respectively)	[174]
	GWAS	Identification of 304 genomic variants that explain 21.2% of CAD heritability with ~80% related to blood vessel morphogenenis, lipid metabolism, NOS and inflammation	[175]
Urinary proteomic biomarkers for CAD	Proteomics	Identification of 15 peptides from urinary proteomics that showed CAD-specific characteristics	[178]
Network analysis explained the underlying mechanism of CAD	Network	Functional CAD network with genetic variants and transcriptomic data	[179]
Generation of CAD-specific regulatory genes and proteins network	Network	Integration of gene–gene networks with protein–protein interactions	[180]
Network analysis revealed shared regulatory pathways with T2D	Network	Genetic regulatory network	[181]

CAS, coronary artery disease; GWAS, genome-wide association study; hCRP, human C-reactive protein; LDL, low-density lipoprotein; NOS, nitric oxide synthase; T2D, type 2 diabetes.

Early detection of CAD is required to perform adequate interventions due to the severity of the disease. Non-invasive diagnosis methods, such as exercise electrocardiogram (ECG), have been widely preferred in clinical settings for suspected CAD patients. These methods have been combined by assessing sociodemographic data, patient description of the pain [165, 166] such as angina and patient age to diagnose obstructive CAD, with this being the most common form of CAD. The less prevalent non-obstructive CAD is also generally diagnosed in patients with angina, and this type of CAD can be accompanied by coronary microvascular dysfunction [167]. In this context, diagnostic tests have been developed to assess this type of microvascular dysfunction to identify patients at risk of future abnormal cardiac events [168]. A number of these tests include the use of ECG for monitoring and interpretation purposes. Although the ECG’s sensitivity has been questioned [169], several computational methods employing artificial intelligence have been developed to significantly increase the sensitivity and accuracy of the ECG [170, 171]. The development of coronary computed tomographic angiography has been implemented to minimize invasive testing whilst improving accuracy of the testing. However, it was shown not to improve clinical outcomes over the early stage of CAD compared to functional testing (ECG, nuclear stress testing or stress echocardiography) [172].

Several studies have amplified the role of genetics in being one of the main risk factors of CAD with earlier studies showing that CAD has a high degree of heritability, especially in male subjects [173, 174]. Recent advances in high-throughput sequencing technologies have allowed for GWAS studies (Table 1) finding more than 300 genetic variants associated with CAD [175, 176]. A further study combined a two-stage GWAS and targeted metabolomics approach to show an association between carbamoyl-phosphate synthase 1 (CPS1) and CAD [177]. In addition, the variant localized to CPS1 was associated with decreased risk of CAD in female subjects. Furthermore, the application of urinary proteomics has been able to find linkages between CKD and CAD by identifying 15 signature peptides of CAD [178].

Several biological networks have been used to explain CAD (Table 1), including a recently generated functional network that integrated genetic variants and transcriptomic data from 600 CAD subjects in the STARTNET consortium [179]. This study successfully uncovered the mechanism of cis- and trans-acting loci in cardiometabolic disease, a precursor of CAD, and showed a gene-regulatory site for blood lipids. A separate study integrated gene-networks with known protein–protein interactions to generate regulatory-gene and protein networks across multiple tissues related to CAD [180]. The networks were combined with known GWAS loci associated with CAD to reveal the disease’s mechanism and propose novel drug targets. An additional study also generated genetic regulatory networks for CAD [181] and identified critical regulatory pathways shared between CAD and T2D. Moreover, this study discovered 15 essential driver genes that showed a high degree of connectivity with known GWAS hits in both CAD and T2D.

### Ischaemic stroke

Ischaemic strokes account for 84% of all stroke events but only slightly fewer deaths than the other primary stroke type, haemorrhagic stroke [182]. Ischaemic strokes occur when a blood vessel leading to the brain is blocked. This can be attributed to several factors and causes include thrombosis, cardioembolism and stenosis. Carotid artery stenosis (CAS) narrows the carotid artery and is responsible for around 7% of ischaemic strokes [183]. A multitude of CAS risk factors, including age, hypertension, diabetes, dyslipidaemia, hypercholesterolaemia and smoking, account for the observed increase in the global burden of CAS, particularly in ageing populations. Middle cerebral artery occlusion (MCAO) refers to the blockage of the middle cerebral artery and is another cause of ischaemic stroke. Standard treatment includes IV thrombolysis, to be administered within hours of the stroke, and intra-arterial therapy, to be sustained over years [184]. In addition to these, aggressive mechanical clot disruption [185] and stenting [186] may be employed if drug treatments fail. This section summarizes the findings of completed studies that have identified different causes and treatments for CAS and MCAO (Table 2).

**Table 2:** Survey of completed studies investigating different causes and treatments for carotid artery stenosis and middle cerebral artery occlusion

Claim	Method	Result	Ref
Carotid artery stenosis (CAS)			
Statins lower LDL and stroke risk	Clinical trial	Atorvastatin lowered LDL compared to placebo (1.9 mmol/L down from 3.3 mmol/L, *P* < 0.001)	[188]
		Atorvastatin reduced stroke risk compared to placebo (11.2% incidence down from 13.1%, *P* = 0.03)	
	Clinical trial	Rosuvastatin lowered LDL by 50% (*P* < 0.001)	[189]
		Rosuvastatin reduced cardiovascular events by 43% (*P* < 0.00001)	
Surgical procedures lower stroke risk	Clinical trial	In asymptomatics, CEA lowered cardiovascular event risk compared to deferred CEA (4.1% down from 10.0%)	[192]
		Greatest net benefit from CEA was identified in those also on lipid-lowering therapy	
	Clinical trial	In asymptomatics, CEA + aspirin/medical management lowered cardiovascular event risk compared to aspirin/medical management alone (5.1% down from 11.0%)	[193]
Antibody action lowers LDL	Clinical trial	Evolocumab lowers LDL by 59% (0.78 mmol/L down from 2.4 mmol/L, *P* < 0.001)	[190]
	Clinical trial	Inclisiran lowers PCSK9 and LDL cholesterol levels (reductions of 35.5–52.6% after two doses, *P* < 0.001) for patients at high cardiovascular risk	[208]
Genomics predicts carotid artery lumen diameter	GWAS	In the Arizona Strong Heart Family Study, genomic loci on chromosomes 7 and 12 were found to be significantly associated with left carotid artery diastolic and systolic lumen diameters	[194]
Genomics predicts moyamoya disease susceptibility	GWAS	Ten genomic loci were identified as significantly linked to moyamoya disease, including previously characterized loci on chromosome 17q25	[195]
	GWAS	In a Japanese cohort, the RNF213 gene on chromosome 17q25 was found to be highly associated with moyamoya disease	[196]
miRNA transcriptomics predicts stroke risk	miRNA association study	In asymptomatics, five miRNAs were identified as significantly overexpressed in patients with disease progression compared to those without	[197]
Biological modelling of CAS is in its infancy	Network	fMRI-based functional network	[198]
	Network	Doppler sonogram-based neural network	[199]
Middle cerebral artery occlusion (MCAO)			
Brain metabolic activity is altered post-MCAO	NMR	In rats, MCAO resulted in increased lactose, alanine, glutamine and GABA and decreased aspartate, glutamate, succinate and creatine due to increased glycolysis and decreased TCA flux	[203]
	MS imaging	In mice, MCAO resulted in increased phosphocreatine, creatine and ceramide. Effects were most pronounced in the caudoputamen and cortex and least pronounced in the hippocampus	[204]
Brain RNA content is altered post-MCAO	RNA-seq	lncRNA-mRNA co-expression network based on rat data, showing lncRNA regulatory changes post MCAO	[202]
	Microarray	Circular RNAs, a lesser-studied class of ncRNA, are differentially expressed in mice post-MCAO	[201]
	RNA-seq	In mice, astrocytes respond to MCAO by upregulation of genes associated with complement and coagulation cascades, scar formation, inflammation, and apoptosis	[200]
		Jak/Stat signalling was upregulated; knocking out STAT3 resulted in increased neuron survival after MCAO	
MCAO induces protein aggregation	MS	In mice, following MCAO, proteins associated with DNA/RNA processing and signal transduction increased in insolubility, indicating protein aggregation	[209]
Neuroprotective drugs mitigate MCAO effects	2DGE/MS	In mice, quercetin mitigated the drop in ICDH, adenosylhomocysteinase, pyruvate kinase, and UCH-L1, and the increase in HSP60 and CRMP-2 protein expression as a result of MCAO	[205]
	2DGE/MS	In rats, resveratrol mitigated the drop in Prx-5, ICDH, ApoA-1 and UCH-L1, and the increase in CRMP-2 as a result of MCAO	[210]

ApoA-1, apolipoprotein A1; CAS, carotid artery stenosis; CEA, carotid endarterectomy; CRMP-2, collapsing response mediator protein 2; fMRI, functional magnetic resonance imaging; GABA, gamma-aminobutyric acid; GWAS, genome-wide association study; HSP60, heat shock protein 60; ICDH, isocitrate dehydrogenase; LDL, low-density lipoprotein; lncRNA, long non-coding RNA; MCAO, middle cerebral artery occlusion; miRNA, microRNA; MS, mass spectrometry; ncRNA, non-coding RNA; NMR, nuclear magnetic resonance, PRX-5, peroxisome assembly factor 5; RNF213, Ring Finger Protein 213; STAT3, signal transducer and activator of transcription 3; TCA, tricarboxylic acid cycle; UCH-L1, ubiquitin carboxy-terminal hydrolase L1; 2DGE, 2D Gel Electrophoresis.

Current diagnosis and risk stratification techniques for CAS include duplex ultrasound, computed tomography angiography, magnetic resonance angiography, digital subtraction angiography and transcranial Doppler (TCD) ultrasound [187]. Of these, TCD is the common technique of choice and has been used extensively to detect microembolic signals and stratification of high- and low-risk carotid lesions. Statins are commonly used to treat CAS by reducing blood LDL levels, thereby decreasing stroke risk, as demonstrated in clinical trials [188, 189]. More recently, antibody action against PCSK9 has also shown the ability to lower LDL levels [190] and have a positive impact on stroke prevention [191]. Finally, surgical procedures such as carotid endarterectomy and stenting are also commonly performed in high-grade stenosis cases, and these procedures confer increased benefits compared to therapeutic solutions alone [192, 193].

Genetic and genomic features in CAS have been markedly less studied than the contribution of diet, smoking and other environmental factors. However, a few studies are noteworthy. For instance, the Strong Heart Family Study, which investigated the heritability of CAS risk among American Indian tribes, found significant genetic linkage on chromosome 7q with left carotid artery lumen diameters in Arizona participants, suggesting candidate genes such as KCND and Kv4 as associated with CAS [194]. GWAS studies have also uncovered susceptibility loci for the related moyamoya disease, which is characterized by CAS [195, 196]; and microRNA biomarkers of CAS progression have been described [197]. Therefore, modelling CAS as an outcome of a biological network model should be considered. CAS has also been modelled as a functional network [198] as well as a NN [199]. In the functional network, fMRI data from 90 pre-specified regions of interest were processed, and correlations in signal intensities formed edges in the network. In the NN, parameters from Doppler sonogram spectroscopy were selected as the input layer. The two models described significant differences in fMRI and Doppler signatures between CAS patients and healthy controls. However, the accuracy of the NN diminished when used to predict CAS severity, and neither of these approaches considers genetic risk factors to CAS progression.

In contrast to CAS research, MCAO research has focused on biological effects post-stroke, and many studies have used mice and rats as a model. MCAO is modelled by anaesthetizing animals and then transient blocking off the middle cerebral artery by ligation or the use of a vascular clip, thereby inducing MCAO. The effects of MCAO on these animals can then be compared to the sham experiment in which animals undergo the same procedure of anaesthesia and exposure of the middle cerebral artery but without occlusion. Recent studies using this method have uncovered biochemical changes on the RNA, protein, and metabolic levels. Furthermore, these models have been useful in characterizing the effects of drugs on mitigating the modifications induced by MCAO. MCAO models have shown differential expression of coding and non-coding transcripts compared to animals undergoing the sham operation. In particular, genes associated with coagulation, migration, scar formation, inflammation and apoptosis were upregulated in mouse astrocytes, whereas cell adhesion, signalling and metabolism genes were downregulated [200]. This suggests a stress response in these cells, which agrees with studies on lncRNA expression in MCAO rats [195] and circular RNA expression in MCAO mice [201]. These studies used microarrays and RNA-seq to find differentially expressed non-coding RNAs known to regulate genes associated with reactive oxygen species, oxidative stress, and apoptosis. A co-expression network of upregulated and downregulated mRNAs and lncRNAs in the MCAO rat further implicates diverse pathways, such as chemokine signalling, Parkinson’s disease, the cell cycle and metabolism [202].

Mass spectrometry and nuclear magnetic resonance studies on MCAO mice and rats have shown metabolic changes post-MCAO. In rats, MCAO increased lactose, alanine, glutamine and GABA compared to the sham procedure rat, whereas aspartate, glutamate, succinate, and creatine decreased [203]. In mice, MCAO accumulates phosphocreatine and ceramide in an ischaemic core, whereas creatine levels decreased [204]. Taken together, this suggests that the restriction of blood flow due to MCAO significantly impacts energy metabolism, resulting in an increase in glycolytic flux and decrease in TCA flux, thus bringing about the observed metabolic changes and gene expression responses to oxidative stress and neuroinflammation. Quercetin and resveratrol are plant-based antioxidants drugs frequently used in Alzheimer’s disease and Parkinson’s disease treatment. Briefly, quercetin works as an inhibitor of acetylcholinesterase and an inhibitor of amyloid-beta filbril formation, whereas resveratrol is an activator of the anti-ageing factor SIRT-1. In experiments with MCAO mice, quercetin mitigated MCAO-related pyruvate kinase decreases as well as decreases in isocitrate dehydrogenase and adenosylhomocysteinase, restoring expression to sham-like levels [205]. Resveratrol also mitigated declines in the latter two proteins in experiments with MCAO rats.

Taken together, the insights from whole omics studies in CAS and MCAO research show that the field would greatly benefit from further work. As we have illustrated, entire omics research in CAS is relatively unexplored. Still, new avenues such as investigating multi-omic responses to drugs and treatments exist and have been more clearly explored for MCAO. There is also real potential for the integration of multiple data types as insightful genomic studies and studies based on imaging and/or physical data have been carried out separately. Current limitations in the field include little to no investigation in personalization of treatment despite the known heterogeneity of symptoms and risk factors for CAS and MCAO; whereas methods for studying blockage of these arteries have not significantly advanced in recent decades.

## Conclusion

The complexity and heterogeneity of CVD strongly suggest that personalized treatments are required depending on the underlying cause that is contributing towards the development and progression of CVD. This includes atherosclerosis, thrombosis, adverse cardiac remodelling, CKD and the gut microbiota. We highlight recent systems biology approaches that have analysed high-throughput omics data to explain these underlying causes further and give examples of how these approaches have contributed significantly towards providing a prognosis for CVD subjects. Such an approach might predict how a myocardial infarction progresses based on a patient’s unique platelet phenotype or predict the development of atherosclerosis through several factors including genetics, behavioural and metabolism-related disorders. The latter of these aforementioned factors can be linked to the composition of the gut microbiome. Hence, we summarized how several bacterial CAGs may affect atherosclerosis by modulating several metabolic pathways in the host. We also assessed how GEMs have enabled us to gain a further understanding of heart-specific metabolic processes through the construction of the *iCardio* GEM and subsequent integration with transcriptomics data to reveal decreased NO and Neu5Ac synthesis as common metabolic markers of heart failure across multiple datasets.

In this review, we also focused on the application of biological networks and association studies in identifying causes and providing treatments for end-stage CVD, namely CAD and stroke, with our chosen examples identifying essential genes for stratifying and treating these two main types of CVD. Future studies should combine all of the fundamental biological mechanisms that underlie the complex pathophysiology of CVD for early prognosis preceding the occurrence of an atherothrombotic event. This could be achieved by integrating current systems biology approaches that have been applied towards explaining the underlying causes of CVD.

We expect future systems biology approaches in cardiovascular research to integrate disease-spanning data from the cardiovascular field with data from other chronic diseases, including the integration of data from CKD and CVD to identify shared commonalities of risk factors and further assess the high comorbidity of these diseases. There is also the need to further understand tissue-specific metabolic crosstalk through integration of models of the heart and other human tissues with the gut microbiota for the simulation of whole-body metabolic functions in health and CVD, with such roadmaps having already been designed for the study of liver-associated diseases [206]. This multi-tissue approach has been limited to date for CVD [207] and future efforts should focus on the generation of a final predictive model incorporating essential metabolic interactions or signalling pathways in each individual tissue/microbiota model that are known to either protect against or contribute towards the development and progression of CVD.

## Authors’ contribution

SD, MA, SL and AB wrote the manuscript, and all authors were involved in the editing and reviewing of the manuscript.

## Supplementary Material

Biographical_Note_bbab061

## Notes

**Stephen Doran** is a PhD student at King’s College London supervised by Prof. Adil Mardinoglu. He is interested in the multi-omics modelling of cardiovascular disease.

**Muhammad Arif** is a PhD student at KTH Royal Institute of Technology, Science for Life Laboratory supervised by Prof. Adil Mardinoglu. He is interested in the multi-omics modelling of cardiovascular disease.

**Simon Lam** is a PhD student at King’s College London supervised by Prof. Adil Mardinoglu. He is interested in the discovery of novel drug targets for effective treatment of Parkinson’s disease.

**Abdulahad Bayraktar** is a PhD student at King’s College London supervised by Prof. Adil Mardinoglu. He is interested in the discovery of novel drug targets for the effective treatment of Alzheimer’s disease.

**Professor Hasan** Türkez currently works at the Department of Medical Biology, Atatürk University. Hasan does research on Genetics, Drug R&D and Nano-Biotechnology.

**Professor Mathias Uhlen** currently works at KTH Royal Institute of Technology Stockholm. His research is focused on protein science, antibody engineering and precision medicine. It ranges from basic research in human and microbial biology to more applied research, including clinical applications in cancer, infectious diseases, cardiovascular diseases, autoimmune diseases and neurobiology.

**Professor Jan Borén** is head of the Institute of Medicine, Sahlgrenska Academy at the University of Gothenburg. His main research interest is lipid metabolism. He has focused on understanding the consequences of and underlying mechanisms that lead to lipid accumulation in the liver, arterial wall and heart, to translate this knowledge into effective treatment.

**Professor Adil Mardinoglu** leads a computational group in the Centre for Host-Microbiome Interactions, King’s College London and also works as a group leader in Science for Life Laboratory, KTH Royal Institute of Technology. His research activities include the generation of the context-specific genome-scale metabolic models for human cell-types including hepatocytes, adipocytes and myocytes as well as certain types of cancer, e.g. liver, prostate, glioblastoma and colon cancers.
