# Supplementary material for: Multi-omics approaches for revealing the complexity of cardiovascular disease
Source: Brief Bioinform. 2021 Mar 17;22(5):bbab061. doi: 10.1093/bib/bbab061 (PMC8425417; doi:10.1093/bib/bbab061)
Supplement: Biographical_Note_bbab061 [file biographical_note_bbab061.docx]

**A biographical note on the authors**

Stephen Doran is a PhD student at King’s College London supervised by Professor Adil Mardinoglu. He is interested in the multi-omics modelling of cardiovascular disease.

Muhammad Arif is a PhD student at KTH Royal Institute of Technology, Science for Life Laboratory supervised by Professor Adil Mardinoglu. He is interested in the multi-omics modelling of cardiovascular disease.

Simon Lam is a PhD student at King’s College London supervised by Professor Adil Mardinoglu. He is interested in the discovery of novel drug targets for effective treatment of Parkinson's disease.

Abdulahad Bayraktar is a PhD student at King’s College London supervised by Professor Adil Mardinoglu. He is interested in the discovery of novel drug targets for effective treatment of Alzheimer's disease.

Prof. Dr. Hasan Türkez currently works at the Department of Medical Biology, Atatürk University. Hasan does research in Genetics, Drug R&D and Nano-Biotechnology.

Professor Mathias Uhlen currently works at KTH Royal Institute of Technology Stockholm. His research is focused on protein science, antibody engineering and precision medicine and ranges from basic research in human and microbial biology to more applied research, including clinical applications in cancer, infectious diseases, cardiovascular diseases, autoimmune diseases and neurobiology.

Professor Jan Borén is Head of the Institute of Medicine, Sahlgrenska Academy at University of Gothenburg. His main research interest is lipid metabolism and he has focused to understand the consequences of and underlying mechanisms that lead to lipid accumulation in the liver, arterial wall and heart, with the goal of translating this knowledge into effective treatment.

Professor Mardinoglu leads a computational group in the Centre for Host-Microbiome Interactions, King’s College London and also works as group leader in Science for Life Laboratory, KTH-Royal Institute of Technology. His research activities include the generation of the context specific genome-scale metabolic models for human cell-types including hepatocytes, adipocytes and myocytes as well as certain types of cancer e.g. liver, prostate, glioblastoma and colon cancers.
